# Absence of a causal link between COVID-19 and deep vein thrombosis: Insights from a bi-directional Mendelian randomisation study

**DOI:** 10.7189/jogh.14.05001

**Published:** 2024-01-12

**Authors:** Mingxuan Li, Lei Xiao, Jiasheng Cai, Kewei Jiang, Yanglei Li, Siqi Li, Qinyue Wang, Wei Wang, Kailei Shi, Haibo Liu

**Affiliations:** 1 Department of Cardiology, Qingpu Branch of Zhongshan Hospital Affiliated to Fudan University, Shanghai, China; 2 Department of Cardiology, Huadong Hospital Affiliated to Fudan University, Shanghai, China; 3 Department of Respiratory Medicine, Huadong Hospital Affiliated to Fudan University, Shanghai, China

## Abstract

**Background:**

Several large-scale observational studies have found deep vein thrombosis (DVT) to be related with coronavirus disease 2019 (COVID-19). However, whether there is a clear causal connection between the two is unknown.

**Methods:**

Our primary analytical method was the inverse variance-weighted (IVW) approach, complemented by the Mendelian randomisation-Egger (MR-Egger) and weighted median methods. We also used MR-Egger to examine the presence of pleiotropy and the Mendelian randomisation pleiotropy residual sum and outlier (MR-PRESSO) approach to analyse for heterogeneity in the data.

**Results:**

We did not observe a direct causal relationship between COVID-19 susceptibility (odds ratio (OR) = 1.023; 95% confidence interval (CI) = 0.828–1.264, standard error (SE) = 0.108, *P* = 0.833), hospitalisation (OR = 1.030; 95% CI = 0.943–1.125, SE = 0.374, *P* = 0.720), severity (OR = 0.994; 95% CI = 0.923–1.071, SE = 0.038, *P* = 0.877), and DVT. The results of the reverse Mendelian randomisation (MR) for DVT and COVID-19 susceptibility exhibited heterogeneity and horizontal pleiotropy. Even after removing outliers, we detected no direct causal relationship between the two (OR = 1.015; 95% CI = 0.954–1.080, SE = 0.032, *P* = 0.630). Similarly, we found no direct causal relationship between DVT and COVID-19 hospitalisation (OR = 0.999; 95% CI = 0.907–1.102, SE = 0.050, *P* = 0.999) or severity (OR = 1.014; 95% CI = 0.893–1.153, SE = 0.065, *P* = 0.826).

**Conclusions:**

In this MR study, we identified no direct causal impact in a European population between DVT and the COVID-19 susceptibility, severity, or hospitalisation.

Coronavirus disease 2019 (COVID-19) has quickly expanded globally since 2019, resulting in billions of infections, millions of fatalities, and a worldwide public health catastrophe [1–4]. COVID-19 is a respiratory infectious disease caused by severe acute respiratory syndrome coronavirus 2 (SARS-CoV-2), primarily transmitted through the respiratory tract, relying on the binding domain of the spike protein and its host receptor, the angiotensin-converting enzyme 2 (ACE2) [5]. Post-infection, most individuals manifest symptoms akin to upper respiratory tract infections, such as fever, fatigue, cough, and myalgia [2,4]. However, patients with concomitant chronic conditions in the cardiovascular, respiratory, digestive, immune, and neoplastic systems are more susceptible to progressing towards respiratory failure, septic shock, and multiple organ failure, presenting serious health hazards [1,2,4,6]. Recent large-scale observational studies have indicated a correlation between COVID-19 infection and deep vein thrombosis (DVT) formation [6–10]. DVT has been reported to occur in roughly 14.8% of COVID-19 patients [9]. Nevertheless, the precise causal link between COVID-19 and its development is unknown.

Mendelian randomisation (MR) is a primary data analysis approach applied in epidemiological aetiological inference [11,12]. It differs from observational studies, which are prone to confounding factors and cannot establish causality definitively, yet is distinct from randomised controlled trials, which demand substantial resources and time. Instead, MR uses existing genome-wide association study data and employs instrumental variables, primarily single nucleotide polymorphisms (SNPs), to investigate causal relationships at the genetic level between variables and outcomes [13–16]. Due to the principle of random genetic inheritance, MR is often referred to as a natural randomised controlled experiment.

In this study, we employed large-scale genome-wide association study data (GWAS) on COVID-19 and DVT to implement a bi-directional MR approach, allowing us to investigate the causal link between COVID-19 and the formation of DVT.

## METHODS

### Study design

We designed a bidirectional MR study to investigate the association between DVT development and COVID-19 susceptibility, hospitalisation, and severity. We first employed COVID-19-associated genetic variations as instrumental variables to analyse COVID-19’s causative influence on the development of DVT. We then used genetic variations associated with DVT as instrumental variables to assess the causative influence of DVT on COVID-19. The criteria for genetic variation selection were as follows [11]: strong association with the exposure, minimal correlation with potential confounding factors, and no direct impact on the outcome.

### Data source

We used two independent GWAS data sets. The data for COVID-19 were obtained from the COVID-19 Host Genetics Initiative V5 [17], which included susceptibility data for 1 683 768 individuals (38 984 infected cases and 1 644 784 controls), hospitalisation data for 1 887 658 individuals (9986 hospitalized patients and 1 877 672 controls), and severity data for 1 388 342 individuals (5101 critically diagnosed respiratory system patients and 1 383 241 controls). We used uninfected individuals as controls. We then sourced GWAS data for DVT from Finland’s FINN-V8 database [18], which comprised 1 303 091 individuals (8077 infected cases and 295 014 controls). All cases were diagnosed through laboratory tests, self-reports, or physician diagnosis (**Table 1**). We did not require ethical approval, as all the data were publicly accessible.

**Table 1:** Data source

Variable	Cases	Controls	Sample size	Year	Population	PubMed ID
COVID-19 susceptibility	38 984	1 644 784	1 683 768	2021	European	32404885
COVID-19 hospitalisation	9986	1 877 672	1 887 658	2021	European	32404885
COVID-19 severity	5101	1 383 241	1 388 342	2021	European	32404885
Deep vein thrombosis	8077	295 014	303 091	2022	European	36653562

### Genetic instrument selection

In our MR analysis, SNPs served as instrumental variables. We first set the genome-wide significance criterion at *P* = 5 × 10^−8^, after which we employed a clustering window size of 5000 kb and a linkage disequilibrium threshold of R^2^<0.001 based on the European 1000 Genomes reference panel. We then excluded palindromic SNPs with intermediate allele frequencies [19–21] and checked for SNPs related to potential confounding factors using the PhenoScanner database [22]. We applied the Mendelian randomisation pleiotropy residual sum and outlier (MR-PRESSO) test to detect potential horizontal pleiotropy [23] and addressed its influence by removing outliers. Lastly, we estimated the F-statistic to assess instrument strength, where F<10 indicates weaker strength [24–26]. All the SNPs used in this study and their F-statistic are presented in Table S1 in the **Online Supplementary Document**.

### Confounding factors related to DVT and COVID-19

The main risk factors associated with DVT formation include cancer, inflammatory bowel disease, systemic lupus erythematosus, and varicose veins [27], while primary risk factors for COVID-19 comprise age, gender, and immune abnormalities [28].

### MR analysis

In the MR analysis, we employed the inverse variance weighted (IVW) method as the principal approach to estimate the causal influence of the exposure on the outcome using a fixed-effects model [12]. We assessed for heterogeneity using the Cochran’s Q test, adopting the IVW random-effects technique was adopted as the primary methodology in cases of significant heterogeneity (*P* < 0.05). As supplementary analyses, we applied the MR-Egger and weighted median methods [29–31]. The primary purpose of using MR-Egger was to detect horizontal pleiotropy. Additionally, we considered the credibility of the weighted median analysis based on the proportion of faulty genetic instruments, aiming for <50% of SNPs as a threshold. Meanwhile, we used the MR-PRESSO test to detect and eliminate any outliers that might produce horizontal pleiotropy before re-evaluating the MR effect [23]. To assess if the causal estimates were driven by a single SNP, we performed a leave-one-out analysis, in which each SNP correlated with the exposure was removed in turn and the IVW analysis was repeated.

We carried out all analyses in R, version 4.2.3. (R Core Team, Vienna, Austria) with the ‘TwoSampleMR’ package (version 0.5.4) being used for IVW, weighted median, and MR-Egger regression methods [32], and the ‘MRPRESSO’ package for the MR-PRESSO analysis.

## RESULTS

In the assessment of the causal relationships between COVID-19 susceptibility, severity, hospitalisation, and DVT, we identified rs643434 and rs505922 associated with thrombosis, and rs11085727 as being associated with systemic lupus erythematosus (SLE) through the PhenoScanner database. However, after removing these SNPs and conducting the MR analysis, we found no significant causal relationships between COVID-19 susceptibility (odds ratio (OR) = 1.023; 95% confidence interval (CI) = 0.828–1.264, standard error (SE) = 0.108, *P* = 0.833), hospitalisation (OR = 1.030; 95% CI = 0.943–1.125, SE = 0.374, *P* = 0.720), severity (OR = 0.994, 95% CI = 0.923–1.071, SE = 0.038, *P* = 0.877), and DVT (**Table2**). Furthermore, in the sensitivity analyses involving Cochran’s Q test, MR-Egger intercept, and MR-PRESSO, we detected no significant heterogeneity, horizontal pleiotropy, or outliers in the results, further supporting the conclusions that there are no significant causal relationships between COVID-19 susceptibility, severity, hospitalisation, and DVT (**Table 3**).

**Table 2:** The casual effect of COVID-19 on deep vein thrombosis

Exposure	Outcome	Number of SNPs	Methods	OR (95% CI)	*P*-value
COVID-19 susceptibility	Deep vein thrombosis	6	IVW	1.02 (0.83–1.26)	0.833
			MR-Egger	0.87 (0.42–1.80)	0.720
			Weighted median	0.98 (0.76–1.27)	0.887
COVID-19 hospitalisation	Deep vein thrombosis	4	IVW	1.03 (0.94–1.13)	0.507
			MR-Egger	1.02 (0.84–1.25)	0.855
			Weighted median	1.04 (0.94–1.16)	0.423
COVID-19 severity	Deep vein thrombosis	7	IVW	0.99 (0.92–1.07)	0.877
			MR-Egger	0.97 (0.79–1.19)	0.773
			Weighted median	1.01 (0.94–1.09)	0.823

SNPs – single nucleotide polymorphisms, OR – odds ratio, CI – confidence interval, IVW – inverse variance weighted, MR – mendelian randomization

**Table 3:** Results of sensitivity analyses

			MR-Egger		
**Exposure**	**Outcome**	**Cochran’s Q test**	**Intercept**	***P*-value**	**MR-PRESSO**
COVID-19 susceptibility	Deep vein thrombosis	0.933	0.013	0.666	0.949
COVID-19 severity	Deep vein thrombosis	0.458	0.002	0.931	0.569
COVID-19 hospitalisation	Deep vein thrombosis	0.072	0.008	0.798	0.121
Deep vein thrombosis	COVID-19 susceptibility	0.005	0.026	0.052	0.006
**After removing the outliers**		0.070	−0.021	0.067	0.065
Deep vein thrombosis	COVID-19 severity	0.264	−0.016	0.437	0.299
Deep vein thrombosis	COVID-19 hospitalisation	0.579	0.006	0.815	0.608

MR-Egger – Mendelian randomisation-Egger, MR-PRESSO – Mendelian randomisation pleiotropy residual sum and outlier

In the assessment of the causal relationships between DVT and COVID-19 susceptibility, severity, and hospitalisation, we identified that the rs11757660 and rs6025 were associated with COVID-19 through the PhenoScanner database, so we removed them from the analysis. Subsequently, after conducting the MR analysis, we found no significant causal relationships between DVT and COVID-19 susceptibility (OR = 0.995; 95% CI = 0.926–1.069, SE = 0.036, *P* = 0.889), hospitalisation (OR = 0.999; 95% CI = 0.907–1.102, SE = 0.050, *P* = 0.999), and severity (OR = 1.014, 95% CI = 0.893–1.153, SE = 0.065, *P* = 0.826) (**Table 4**).

**Table 4:** The casual effect of deep vein thrombosis on COVID-19

Exposure	Outcome	Number of SNPs	Methods	OR (95% CI)	*P*-value
Deep vein thrombosis	COVID-19 susceptibility	11	IVW	1.00 (0.93–1.07)	0.889
			MR-Egger	1.14 (1.00–1.31)	0.085
			Weighted median	0.99 (0.92–1.06)	0.788
			IVW (random effects)	1.00 (0.93–1.07)	0.889
**After removing the outliers**		9	IVW	1.02 (0.95–1.08)	0.630
			MR-Egger	1.13 (1.01–1.27)	0.064
			Weighted median	0.99 (0.92–1.07)	0.842
Deep vein thrombosis	COVID-19 hospitalisation	11	IVW (random effects)	1.00 (0.91–1.10)	0.999
			MR-Egger	1.09 (0.87–1.36)	0.486
			Weighted median	0.97 (0.86–1.10)	0.641
Deep vein thrombosis	COVID-19 severity	12	IVW	1.01 (0.89–1.15)	0.826
			MR-Egger	0.98 (0.74–1.31)	0.908
			Weighted median	1.10 (0.93–1.31)	0.256

SNPs – single nucleotide polymorphisms, OR – odds ratio, CI – confidence interval, IVW – inverse variance weighted, MR – Mendelian randomisation

In the sensitivity analyses, we observed heterogeneity and horizontal pleiotropy in the MR analysis between DVT and COVID-19 susceptibility (Cochran’s Q = 0.005, MR-PRESSO = 0.006). We conducted further sensitivity analyses after removing the outliers rs4752927 and rs62350309 and found no heterogeneity and horizontal pleiotropy (Cochran’s Q > 0.05 MR-PRESSO>0.05). We performed the MR analysis again and still found no significant causal relationship (OR = 1.015; 95% CI = 0.954–1.080, SE = 0.032, *P* = 0.630). After conducting Cochran’s Q, MR-Egger intercept, and MR-PRESSO tests for the other two groups, we found no significant heterogeneity, horizontal pleiotropy, or outliers (**Table 3**).

## DISCUSSION

In this study, we used a large-scale GWAS databases from the COVID-19 Host Genetics Initiative and the Finnish database to investigate the causal associations between DVT and COVID-19 susceptibility, severity, and hospitalisation. Our findings demonstrate that there is no direct causal link between COVID-19 susceptibility, severity, or hospitalisation with the formation of DVT.

In early 2020, a clinical study from Hamburg, Germany, conducted post-mortem examinations on patients who died from COVID-19 and found that most had concurrent DVT [7]. Additionally, a cross-sectional study from Wuhan examined 48 critically ill COVID-19 patients and detected vein thrombosis in the lower extremities (including femoral, popliteal, posterior tibial, fibular, and calf muscular veins) in 41 individuals (85.4%) [8]. Suh et al. [9] also conducted a meta-analysis on the incidence of blood clots in COVID-19 patients, assessing a total of 27 studies comprising 3342 COVID-19 patients; they found that overall occurrence rates of pulmonary embolism and DVT to be 16.5% (95% CI = 11.6–2.9) and 14.8% (95% CI = 8.5–24.5), respectively. Another systematic review and meta-analysis found vein thromboembolism incidence rates of 7.9% among non-intensive care unit patients and 22.7% among intensive care unit patients [10]. Despite multiple observational studies finding an association between COVID-19 and DVT, to the best of our knowledge, no MR study has been reported on the relationship between COVID-19 and DVT. Here we used SNPs from two large-scale GWAS databases as instrumental variables. We screened the initial SNPs and conducted sensitivity analysis to eliminate unqualified ones to ensure that SNPs finally included in this study met the three core assumptions of MR. The F-values of the final SNPs were all greater than 10, demonstrating their reliability and indicating that they are not weak instrumental variables and will not be affected by weak instrumental bias, and they can well explain the degree of variation of DVT and COVID-19. After using multiple MR methods, including IVW, MR-Egger, and weighted median, the results of the three analyses do not support a direct causal relationship between DVT and COVID-19 in the European population. Our findings are inconsistent with those of past observational studies, and we suspect that previously reported epidemiological associations may be mediated by some factor or unmeasured confounders [6].

Based on published research, we hypothesise that SARS-CoV-2 may intervene in thrombus formation through several pathways. The first is dysfunction of the renin-angiotensin-aldosterone system (RAAS). Here, SARS-CoV-2 can directly bind to ACE2 [33], leading to an imbalance in the conversion of angiotensin 2 to angiotensin 1. This results in an increase in angiotensin 2 and a decrease in angiotensin 1, which can exacerbate inflammation, oxidative stress, endothelial dysfunction, and promote coagulation [33-37]. Conversely, angiotensin 1–7 inhibits platelet activation and thrombus formation by binding to Mars receptors on endothelial cells, leading to the production of nitric oxide and prostaglandins [38].

The second is endothelial injury and dysfunction. Apart from RAAS dysfunction-induced endothelial injury, ACE2 receptors are widely expressed in endothelial cells. When SARS-CoV-2 invades the body, it replicates in endothelial cells, causing cellular damage and apoptosis, thereby reducing anti-thrombotic activity [39,40]. Furthermore, elevated pro-inflammatory cytokines such as IL-6, IL-1β, and monocyte chemoattractant protein 1 (MCP-1) in COVID-19 patients affect endothelial function and integrity [40,41].

The third is the Von Willebrand factor (vWF) activation. Reports indicate that COVID-19 patients exhibit significantly higher vWF activity and concentration compared to normal controls, and that vWF is a procoagulant factor [42]. When endothelial cells are damaged, vWF is released from the subendothelium [43], facilitating platelet adhesion and aggregation through binding to platelet GPIb-IX complex, subendothelial collagen, and GPIIb-IIIa, thereby promoting thrombus formation.

The fourth is pathway is through cytokine storm: in COVID-19, the cytokine storm is characterised by an excessive production of pro-inflammatory cytokines such as IL-6, IL-1, IL-18, and granulocyte-macrophage colony-stimulating factor (GM-CSF), particularly in severe instances [40,41,44]. These pro-inflammatory compounds can contribute to thrombus formation by a variety of processes, including neutrophil activation, endothelial cell activation, and inflammasome activation, overexpression of adhesion molecules such as P- and E-selectin, prolonging of clot lysis time, and enhanced platelet activity [45–47].

The fifth potential pathway are neutrophil extracellular traps (NETs). When neutrophils are activated, they release NETs to capture platelets and clotting factors [48]. Moreover, NETs participate in various coagulation pathways, promoting the formation of thrombin [49]. The interaction between platelets and NET components leads to the formation of immune thrombi [50].

The last pathway is the macrophage activation syndrome (MAS). Antigen-presenting cells get activated in response to SARS-CoV-2 invasion, triggering an inflammatory response and raising immunological markers, including IL-6. Previous studies have shown that elevated IL-6 levels cause natural killer (NK) cells to perform less cytolytically [51]. When activated antigen presenting cells cannot be lysed by NK cells, MAS is thought to occur. Through the aforementioned cascade, an ongoing interaction between innate and adaptive immune cells takes place, which promotes the production of thrombus and cytokine storms [52]. However, owing to the constraints inherent to a MR study, it is unclear that the extent of adaptive immune responses and dysregulation of blood clotting mechanism.

This study has some limitations. We cannot rule out the potential of not discovering relationships owing to current sample size. Second, even after adjusting for the influence of confounding factors on the results, it is still not completely removed due to limitations of the study method. Furthermore, we did not investigate if the connection between COVID-19 and DVT is mediated by any intermediary factors, which we will address in future research. Finally, the GWAS data used in this study is limited to people of European heritage, thus the findings may not be applicable to other racial or ethnic groups.

## CONCLUSION

In this MR study, we found no direct causal impact in European population between DVT and the severity of COVID-19, hospitalisation, or susceptibility.

## Additional material


Online Supplementary Document

## References

[1] Jin YH Zhan QY Peng ZY Ren XQ Yin XT Cai L Chemoprophylaxis, diagnosis, treatments, and discharge management of COVID-19: An evidence-based clinical practice guideline (updated version). Mil Med Res. 2020;7:41. 10.1186/s40779-020-00270-8 32887670 PMC7472403

[2] Asselah T Durantel D Pasmant E Lau G Schinazi RF COVID-19: Discovery, diagnostics and drug development. J Hepatol. 2021;74:168–84. 10.1016/j.jhep.2020.09.031 33038433 PMC7543767

[3] Davis HE McCorkell L Vogel JM Topol EJ Long COVID: major findings, mechanisms and recommendations. Nat Rev Microbiol. 2023;21:133–46. 10.1038/s41579-022-00846-2 36639608 PMC9839201

[4] Umakanthan S Sahu P Ranade AV Bukelo MM Rao JS Abrahao-Machado LF Origin, transmission, diagnosis and management of coronavirus disease 2019 (COVID-19). Postgrad Med J. 2020;96:753–8. 32563999 10.1136/postgradmedj-2020-138234 PMC10016932

[5] Buchrieser J Dufloo J Hubert M Monel B Planas D Rajah MM Syncytia formation by SARS-CoV-2-infected cells. EMBO J. 2020;39:e106267. 10.15252/embj.2020106267 33051876 PMC7646020

[6] Brito-Dellan N Tsoukalas N Font C Thrombosis, cancer, and COVID-19. Support Care Cancer. 2022;30:8491–500. 10.1007/s00520-022-07098-z 35567609 PMC9106567

[7] Wichmann D Sperhake JP Lutgehetmann M Steurer S Edler C Heinemann A Autopsy Findings and Venous Thromboembolism in Patients With COVID-19: A Prospective Cohort Study. Ann Intern Med. 2020;173:268–77. 10.7326/M20-2003 32374815 PMC7240772

[8] Ren B Yan F Deng Z Zhang S Xiao L Wu M Extremely High Incidence of Lower Extremity Deep Venous Thrombosis in 48 Patients With Severe COVID-19 in Wuhan. Circulation. 2020;142:181–3. 10.1161/CIRCULATIONAHA.120.047407 32412320

[9] Suh YJ Hong H Ohana M Bompard F Revel MP Valle C Pulmonary Embolism and Deep Vein Thrombosis in COVID-19: A Systematic Review and Meta-Analysis. Radiology. 2021;298:E70–80. 10.1148/radiol.2020203557 33320063 PMC7745997

[10] Nopp S Moik F Jilma B Pabinger I Ay C Risk of venous thromboembolism in patients with COVID-19: A systematic review and meta-analysis. Res Pract Thromb Haemost. 2020;4:1178–91. 10.1002/rth2.12439 33043231 PMC7537137

[11] Emdin CA Khera AV Kathiresan S Mendelian Randomization. JAMA. 2017;318:1925–6. 10.1001/jama.2017.17219 29164242

[12] Burgess S Butterworth A Thompson SG Mendelian randomization analysis with multiple genetic variants using summarized data. Genet Epidemiol. 2013;37:658–65. 10.1002/gepi.21758 24114802 PMC4377079

[13] Boef AG Dekkers OM le Cessie S Mendelian randomization studies: a review of the approaches used and the quality of reporting. Int J Epidemiol. 2015;44:496–511. 10.1093/ije/dyv071 25953784

[14] Jansen H Samani NJ Schunkert H Mendelian randomization studies in coronary artery disease. Eur Heart J. 2014;35:1917–24. 10.1093/eurheartj/ehu208 24917639

[15] Kennedy OJ Pirastu N Poole R Fallowfield JA Hayes PC Grzeszkowiak EJ Coffee Consumption and Kidney Function: A Mendelian Randomization Study. Am J Kidney Dis. 2020;75:753–61. 10.1053/j.ajkd.2019.08.025 31837886

[16] Davies NM Holmes MV Davey Smith G Reading Mendelian randomisation studies: a guide, glossary, and checklist for clinicians. BMJ. 2018;362:k601. 10.1136/bmj.k601 30002074 PMC6041728

[17] Initiative C-HG The COVID-19 Host Genetics Initiative, a global initiative to elucidate the role of host genetic factors in susceptibility and severity of the SARS-CoV-2 virus pandemic. Eur J Hum Genet. 2020;28:715–8. 10.1038/s41431-020-0636-6 32404885 PMC7220587

[18] Kurki MI Karjalainen J Palta P Sipila TP Kristiansson K Donner KM FinnGen provides genetic insights from a well-phenotyped isolated population. Nature. 2023;613:508–18. 10.1038/s41586-022-05473-8 36653562 PMC9849126

[19] Huang D Lin S He J Wang Q Zhan Y Association between COVID-19 and telomere length: A bidirectional Mendelian randomization study. J Med Virol. 2022;94:5345–53. 10.1002/jmv.28008 35854470 PMC9349767

[20] Peng H Wu X Xiong S Li C Zhong R He J Gout and susceptibility and severity of COVID-19: A bidirectional Mendelian randomization analysis. J Infect. 2022;85:e59–61. 10.1016/j.jinf.2022.05.042 35724757 PMC9212781

[21] Zhou X Yu L Wang L Xiao J Sun J Zhou Y Alcohol consumption, blood DNA methylation and breast cancer: a Mendelian randomisation study. Eur J Epidemiol. 2022;37:701–12. 10.1007/s10654-022-00886-1 35708873 PMC9329409

[22] Kamat MA Blackshaw JA Young R Surendran P Burgess S Danesh J PhenoScanner V2: an expanded tool for searching human genotype-phenotype associations. Bioinformatics. 2019;35:4851–3. 10.1093/bioinformatics/btz469 31233103 PMC6853652

[23] Verbanck M Chen CY Neale B Do R Detection of widespread horizontal pleiotropy in causal relationships inferred from Mendelian randomization between complex traits and diseases. Nat Genet. 2018;50:693–8. 10.1038/s41588-018-0099-7 29686387 PMC6083837

[24] Palmer TM Lawlor DA Harbord RM Sheehan NA Tobias JH Timpson NJ Using multiple genetic variants as instrumental variables for modifiable risk factors. Stat Methods Med Res. 2012;21:223–42. 10.1177/0962280210394459 21216802 PMC3917707

[25] Levin MG Judy R Gill D Vujkovic M Verma SS Bradford Y Genetics of height and risk of atrial fibrillation: A Mendelian randomization study. PLoS Med. 2020;17:e1003288. 10.1371/journal.pmed.1003288 33031386 PMC7544133

[26] Gill D Efstathiadou A Cawood K Tzoulaki I Dehghan A Education protects against coronary heart disease and stroke independently of cognitive function: evidence from Mendelian randomization. Int J Epidemiol. 2019;48:1468–77. 10.1093/ije/dyz200 31562522 PMC6857750

[27] Stubbs MJ Mouyis M Thomas M Deep vein thrombosis. BMJ. 2018;360:k351. 10.1136/bmj.k351 29472180

[28] Gao YD Ding M Dong X Zhang JJ Kursat Azkur A Azkur D Risk factors for severe and critically ill COVID-19 patients. Allergy. 2021;76:428–55. 10.1111/all.14657 33185910

[29] Bowden J Del Greco MF Minelli C Davey Smith G Sheehan NA Thompson JR Assessing the suitability of summary data for two-sample Mendelian randomization analyses using MR-Egger regression: the role of the I2 statistic. Int J Epidemiol. 2016;45:1961–1974. 10.1093/ije/dyw220 27616674 PMC5446088

[30] Bowden J Davey Smith G Haycock PC Burgess S Consistent Estimation in Mendelian Randomization with Some Invalid Instruments Using a Weighted Median Estimator. Genet Epidemiol. 2016;40:304–14. 10.1002/gepi.21965 27061298 PMC4849733

[31] Burgess S Thompson SG Interpreting findings from Mendelian randomization using the MR-Egger method. Eur J Epidemiol. 2017;32:377–89. 10.1007/s10654-017-0255-x 28527048 PMC5506233

[32] Hemani G Zheng J Elsworth B Wade KH Haberland V Baird D The MR-Base platform supports systematic causal inference across the human phenome. eLife. 2018;7:e34408. 10.7554/eLife.34408 29846171 PMC5976434

[33] Verdecchia P Cavallini C Spanevello A Angeli F The pivotal link between ACE2 deficiency and SARS-CoV-2 infection. Eur J Intern Med. 2020;76:14–20. 10.1016/j.ejim.2020.04.037 32336612 PMC7167588

[34] Turner AJ Hiscox JA Hooper NM ACE2: from vasopeptidase to SARS virus receptor. Trends Pharmacol Sci. 2004;25:291–4. 10.1016/j.tips.2004.04.001 15165741 PMC7119032

[35] Zhang H Penninger JM Li Y Zhong N Slutsky AS Angiotensin-converting enzyme 2 (ACE2) as a SARS-CoV-2 receptor: molecular mechanisms and potential therapeutic target. Intensive Care Med. 2020;46:586–90. 10.1007/s00134-020-05985-9 32125455 PMC7079879

[36] Kuba K Imai Y Penninger JM Multiple functions of angiotensin-converting enzyme 2 and its relevance in cardiovascular diseases. Circ J. 2013;77:301–8. 10.1253/circj.CJ-12-1544 23328447

[37] Patel VB Zhong JC Grant MB Oudit GY Role of the ACE2/Angiotensin 1-7 Axis of the Renin-Angiotensin System in Heart Failure. Circ Res. 2016;118:1313–26. 10.1161/CIRCRESAHA.116.307708 27081112 PMC4939482

[38] Santos RAS Sampaio WO Alzamora AC Motta-Santos D Alenina N Bader M The ACE2/Angiotensin-(1-7)/MAS Axis of the Renin-Angiotensin System: Focus on Angiotensin-(1-7). Physiol Rev. 2018;98:505–53. 10.1152/physrev.00023.2016 29351514 PMC7203574

[39] Targosz-Korecka M Kubisiak A Kloska D Kopacz A Grochot-Przeczek A Szymonski M Endothelial glycocalyx shields the interaction of SARS-CoV-2 spike protein with ACE2 receptors. Sci Rep. 2021;11:12157. 10.1038/s41598-021-91231-1 34108510 PMC8190434

[40] Bonaventura A Vecchie A Dagna L Martinod K Dixon DL Van Tassell BW Endothelial dysfunction and immunothrombosis as key pathogenic mechanisms in COVID-19. Nat Rev Immunol. 2021;21:319–29. 10.1038/s41577-021-00536-9 33824483 PMC8023349

[41] McFadyen JD Stevens H Peter K The Emerging Threat of (Micro)Thrombosis in COVID-19 and Its Therapeutic Implications. Circ Res. 2020;127:571–87. 10.1161/CIRCRESAHA.120.317447 32586214 PMC7386875

[42] Goshua G Pine AB Meizlish ML Chang CH Zhang H Bahel P Endotheliopathy in COVID-19-associated coagulopathy: evidence from a single-centre, cross-sectional study. Lancet Haematol. 2020;7:e575–82. 10.1016/S2352-3026(20)30216-7 32619411 PMC7326446

[43] Leebeek FW Eikenboom JC Von Willebrand’s Disease. N Engl J Med. 2016;375:2067–80. 10.1056/NEJMra1601561 27959741

[44] Mehta P McAuley DF Brown M Sanchez E Tattersall RS Manson JJ COVID-19: consider cytokine storm syndromes and immunosuppression. Lancet. 2020;395:1033–4. 10.1016/S0140-6736(20)30628-0 32192578 PMC7270045

[45] Abou-Ismail MY Diamond A Kapoor S Arafah Y Nayak L The hypercoagulable state in COVID-19: Incidence, pathophysiology, and management. Thromb Res. 2020;194:101–15. 10.1016/j.thromres.2020.06.029 32788101 PMC7305763

[46] Savla SR Prabhavalkar KS Bhatt LK Cytokine storm associated coagulation complications in COVID-19 patients: Pathogenesis and Management. Expert Rev Anti Infect Ther. 2021;19:1397–413. 10.1080/14787210.2021.1915129 33832398 PMC8074652

[47] Di Salvo E Di Gioacchino M Tonacci A Casciaro M Gangemi S Alarmins, COVID-19 and comorbidities. Ann Med. 2021;53:777–85. 10.1080/07853890.2021.1921252 34042528 PMC8168739

[48] Stark K Philippi V Stockhausen S Busse J Antonelli A Miller M Disulfide HMGB1 derived from platelets coordinates venous thrombosis in mice. Blood. 2016;128:2435–49. 10.1182/blood-2016-04-710632 27574188 PMC5147023

[49] Zhu Y Chen X Liu X NETosis and Neutrophil Extracellular Traps in COVID-19: Immunothrombosis and Beyond. Front Immunol. 2022;13:838011. 10.3389/fimmu.2022.838011 35309344 PMC8924116

[50] Jing H Wu X Xiang M Liu L Novakovic VA Shi J Pathophysiological mechanisms of thrombosis in acute and long COVID-19. Front Immunol. 2022;13:992384. 10.3389/fimmu.2022.992384 36466841 PMC9709252

[51] Crayne CB Albeituni S Nichols KE Cron RQ The Immunology of Macrophage Activation Syndrome. Front Immunol. 2019;10:119. 10.3389/fimmu.2019.00119 30774631 PMC6367262

[52] Hanff TC Mohareb AM Giri J Cohen JB Chirinos JA Thrombosis in COVID-19. Am J Hematol. 2020;95:1578–89. 10.1002/ajh.25982 32857878 PMC7674272

