# Supplementary material for: Absence of a causal link between COVID-19 and deep vein thrombosis: Insights from a bi-directional Mendelian randomisation study
Source: J Glob Health. 2024 Jan 12;14:05001. doi: 10.7189/jogh.14.05001 (PMC10786189; doi:10.7189/jogh.14.05001)
Supplement: Online Supplementary Document [file jogh-14-05001-s001.pdf]

Supplementary table 1: The parameters of all SNPs in this study

Exposure	Outcome	SNP number	SNP	BETA	SE	Sample size	MAF	F value
COVID-19 susceptibility	deep vein thrombosis	6	rs10936744	-0.063	0.010	1683768	0.359	39.368
			rs12482060	0.062	0.011	1683768	0.338	34.646
			rs17078348	0.092	0.016	1683768	0.100	32.494
			rs2271616	0.156	0.015	1683768	0.118	107.425
			rs4971066	-0.077	0.013	1683768	0.178	32.816
			rs757405	0.069	0.011	1683768	0.291	40.859
COVID-19 hospitalization	deep vein thrombosis	4	rs13050728	-0.168	0.020	1887658	0.347	69.550
			rs2109069	0.151	0.020	1887658	0.323	57.779
			rs2660	0.116	0.019	1887658	0.310	35.972
			rs35081325	0.488	0.032	1887658	0.081	240.128
COVID-19 severity	deep vein thrombosis	7	rs111837807	0.295	0.043	1388342	0.100	47.444
			rs13050728	-0.200	0.029	1388342	0.337	49.097
			rs2109069	0.257	0.028	1388342	0.329	83.585
			rs2237698	0.237	0.040	1388342	0.090	35.608
			rs2384074	0.198	0.028	1388342	0.324	49.383
			rs35081325	0.626	0.045	1388342	0.075	197.982
			rs77534576	0.460	0.075	1388342	0.035	37.636
deep vein thrombosis	COVID-19 susceptibility	11	rs13377102	-0.233	0.027	303091	0.110	74.353
			rs1799963	0.796	0.088	303091	0.005	81.974
			rs2066865	0.188	0.017	303091	0.301	121.962
			rs2289252	0.204	0.016	303091	0.424	161.634
			rs4752927	0.124	0.020	303091	0.205	39.595
			rs6060237	0.166	0.021	303091	0.163	61.884

Exposure	Outcome	SNP number	SNP	BETA	SE	Sample size	MAF	F value
			rs62350309	-0.160	0.028	303091	0.102	33.609
			rs7135039	0.094	0.017	303091	0.352	31.145
			rs76151810	0.158	0.029	303091	0.078	29.860
			rs9863058	-0.100	0.017	303091	0.368	35.740
			rs9865118	0.093	0.016	303091	0.494	33.107
deep vein thrombosis	COVID-19 hospitalization	11	rs13377102	-0.233	0.027	303091	0.110	74.353
			rs1799963	0.796	0.088	303091	0.005	81.974
			rs2066865	0.188	0.017	303091	0.301	121.962
			rs2289252	0.204	0.016	303091	0.424	161.634
			rs4752927	0.124	0.020	303091	0.205	39.595
			rs6060237	0.166	0.021	303091	0.163	61.884
			rs62350309	-0.160	0.028	303091	0.102	33.609
			rs7135039	0.094	0.017	303091	0.352	31.145
			rs76151810	0.158	0.029	303091	0.078	29.860
			rs9863058	-0.100	0.017	303091	0.368	35.740
			rs9865118	0.093	0.016	303091	0.494	33.107
deep vein thrombosis	COVID-19 severity	12	rs13377102	-0.233	0.027	303091	0.110	74.353
			rs1799963	0.796	0.088	303091	0.005	81.974
			rs192669930	0.612	0.101	303091	0.005	36.550
			rs2066865	0.188	0.017	303091	0.301	121.962
			rs2289252	0.204	0.016	303091	0.424	161.634
			rs4752927	0.124	0.020	303091	0.205	39.595
			rs6060237	0.166	0.021	303091	0.163	61.884
			rs62350309	-0.160	0.028	303091	0.102	33.609

**Exposure**	**Outcome**	**SNP number**	**SNP**	**BETA**	**SE**	**Sample size**	**MAF**	**F value**
			rs7135039	0.094	0.017	303091	0.352	31.145
			rs76151810	0.158	0.029	303091	0.078	29.860
			rs9863058	-0.100	0.017	303091	0.368	35.740
			rs9865118	0.093	0.016	303091	0.494	33.107
